# Risky Lives? Self-Directed Violence and Violence From Others Among Young People Not in Education, Employment, or Training (NEET)

**DOI:** 10.3389/fpubh.2022.904458

**Published:** 2022-07-07

**Authors:** Siri Havas Haugland, Tonje Holte Stea

**Affiliations:** ^1^Department of Psychosocial Health, University of Agder, Kristiansand, Norway; ^2^Department of Health and Nursing Science, University of Agder, Kristiansand, Norway

**Keywords:** not in education, employment, or training (NEET), self-harm, suicide-attempt, violence

## Abstract

Self-directed violence and violence from others comprise a major public health concern in youth. However, we lack knowledge about the prevalence of violent experiences among young people that are not in education, employment, or training (NEET), whether violent occurrences are similar among girls and boys, and whether violence differs between NEET youths and young students. This cross-sectional study compared the occurrences of self-directed violence (suicide attempts and self-harm) and exposure to violence from others (violent threats, beaten without visible marks, or injured due to violent events) between 96 NEET youth and 384 age- and sex-matched upper secondary school students (16–21 years). Suicide attempts were reported by 45.2% of NEET girls and 18.8% of schoolgirls (*p* < 0.001), but no significant difference was found between NEET boys (17.6%) and schoolboys (13.1%). Self-harm was reported by 78.9% of NEET girls and 33.9% of schoolgirls (*p* < 0.001). Self-harm was less prevalent among boys; it was reported by 34.6% of NEET boys and 21.8% of schoolboys (*p* = 0.056). A multivariable logistic regression analysis, adjusted for parental education, showed that, compared to schoolgirls, NEET girls had much higher odds ratios (ORs) for both self-directed violence and violence from others with OR ranging from 4.39; CI 1.96–9.85 to 7.68; CI 3.20–18.41. The risk of injury due to violent events was higher among NEET boys (OR: 3.23, 95%CI: 1.21–8.62) compared to schoolboys. Our findings highlighted the importance of including NEET individuals in studies on violence and emphasized the importance of psychosocial health services for young people marginalized from education and the labor market.

## Introduction

Self-directed violence constitutes a major public health issue among youth and young adults 10–29 years. The World Health Organization 67 (WHO) has estimated that self-harm causes 256 180 deaths globally per year among this age group (1). Estimations also show that, last year, globally, two out of three children under 18 years old experienced violence from others (2). Exposure to violence is a serious threat to both physical and mental health. To prevent life-long consequences, it is crucial to identify vulnerable groups and life-situations. Adverse outcomes of violent experiences may be both acute and long-lasting; they include physical injuries, mental health problems (e.g., depression, anxiety, and post-traumatic stress disorder), social, emotional, and behavioral problems, suicide, disabilities, poor educational attainment, reduced cognitive ability, and an increased risk of non-communicable diseases (3, 4). Social exclusion or marginalization can increase the risk of exposure to violence in certain groups. However, we lack studies that have investigated the link between disengagement from employment/education and violence (3).

The proportion of young people that are not engaged in education, employment, or training (NEET) is a rising concern in Europe (5, 6). Although NEET individuals comprise a heterogenous group (6), disengagement from social institutions, such as education and the labor market, is associated with a range of life difficulties (7), including impaired mental health (8–10). Some studies have suggested that NEET individuals have reported more advanced stages of mental illness than their peers (11), and they reported more self-directed violence, such as suicidal behaviors (8, 12, 13). However, the scientific evidence that shows increased rates of self-directed violence among NEETS compared to the general population is scarce, and the findings are somewhat inconsistent (14). In the general population, self-directed violence in youth has been identified as a major public health concern (1) that warrants more knowledge about possible risk factors. Suicidal behavior among males and females differs and this known as the “the gender paradox in suicide,” where non-fatal suicidal behavior is more common among females, but males are overrepresented among those who died by suicide (15, 16). A study by Chan et al. also reported that the risk of self-directed violence among unemployed youth was different between the sexes: males were more likely to die by suicide than females (17). However, results from other studies in the general population have shown that self-harm and suicide attempts had increased over time more among women than among men (18, 19). Moreover, a population-level record-linkage study (20) confirmed that unemployment increased the risk of suicide among males, and not being in the labor force (e.g., students, retirees, voluntary inactive, permanently unable to work) increased the risk of suicide among females, but not males.

The etiology of these associations is complex. It remains unclear whether mental health problems is a cause or a result of marginalization from education and the labor market. Findings from Baggio et al. (21) suggested that marginalization from education, employment, and training was a consequence, rather than a cause, of mental health problems and substance use. A longitudinal study by Mars et al. (22) also found that suicidal self-harm at age 16 years was associated with poorer educational and occupational outcomes at age 19, and with the risk of being NEET at age 19 years. Lee et al. (16) also found that older adolescent who died by suicide were more likely to be NEETs, but this group also had a higher prevalence of formally diagnosed mental illness than the general population. However, Cunningham et al. (20) found that marginalization from the labor market was also associated with self-harm and suicide among individuals without prior mental health problems. Those results were supported by a literature review on unemployment and psychological distress among young adults (23), which reported that the risk of suicide attempts increased with unemployment among young adults, even after accounting for the initial mental health status and other confounding factors.

Consequently, it remains unclear whether the reported increase in self-directed violence is related to an increase in marginalization from education and the labor market. Studies have shown inconsistent findings on the relationship between periods of economic hardship (e.g., the crisis of 2008) and the prevalence of self-harm or suicides. A longitudinal multicenter study from the UK (24) found that self-harm increased in areas with greater rises in unemployment. However, a study from Iceland (25) did not find an increase in hospital attendance due to self-harm or suicide attempts following the Icelandic economic collapse in 2008.

Adverse life experiences, such as violence from others, were found to impact educational attainment and employment (26, 27). A global systematic review and meta-analysis of the relationships between violence in childhood and educational outcomes (28) consistently showed that all forms of violence had a significant impact on various educational outcomes, including school absences, dropping-out, graduation, and academic achievement. Those results were confirmed in a study that showed a strong, consistent gradient relationship between low socioeconomic status (measured as a low educational level and receiving welfare benefits) and the frequency of adverse childhood experiences (29). The WHO pointed out that females are particularly vulnerable to exposure to violence (3). However, a study that investigated social and health related problems among young adult NEETs did not find any significant differences in exposure to violence between males and females, except sexual violence (30). However, despite findings that experiences of violence or self-directed violence were associated with marginalization from education and the labor market, most larger health studies that focused on self-directed violence or violence were carried out in schools, such as the Child and Adolescent Self-Harm in Europe study and the Health Behavior in School-aged Children survey (31, 32).

Due to the lack of knowledge about the risk of exposure to violence among NEET individuals, the present study aimed to compare the prevalence of self-directed violence (self-harm and suicide attempts) and violence from others between a selection of vulnerable young NEET individuals and their peers that attended upper-level secondary schools.

## Materials and Methods

The present study was based on two cross-sectional studies conducted in southern Norway. One study was called “Health, living conditions, and lifestyles among young people who are not in education, employment, or training” (the HELLAS study), and it targeted adolescents registered as NEETs. The other was called the Young Data study (Ungdata), and it targeted adolescents that attended upper-level secondary schools. Participants in the HELLAS study was the main target group in our study. Participants in the Young Data study were selected to form an age- and sex-matched reference group. Both studies were conducted in 2016. All recruited participants received oral and written information about the studies. For adolescents under the age of 16, the parents also received written information about the studies. Participation was voluntary, and both adolescents and parents could refuse participation or withdraw at any time during or after data collection. After written consent was obtained, all participants were instructed to complete an online, self-report questionnaire. The questionnaire required approximately 20–45 min to complete. All responses were treated anonymously, and ethical approval and research clearance were provided by the South-East Regional Committee for Medical and Health Research Ethics (REK case no. 2015/2431).

### Samples

#### The HELLAS Study

NEETs are a highly heterogenic group that includes both vulnerable and non-vulnerable young people. The European Foundation for the Improvement of Living and Working Conditions (Eurofound) has suggested that the NEET-group can be categorized into five groups: the conventionally unemployed, the unavailable, the disengaged, voluntary NEETs and opportunity seekers (6). In Norway, all young people 16–21 years of age, who are entitled to training but not enrolled in public schools or paid employment, are registered in a database by an advisory service. In this process the NEETs are categorized into different sub-groups by the advisory service that allows the identification of a more vulnerable target group that require follow-up, support or interventions. Voluntary NEETs or youths in private school are typically not defined to be in this vulnerable group. This database provided an opportunity for the present study to target and recruit the more vulnerable group with a higher risk of marginalization among the Norwegian NEETS. At the time of data collection, 685 NEET youths, categorized as vulnerable to marginalization, were registered with the follow-up services of the southern region of Norway. These NEET individuals received letters with information about the study and an invitation to participate. In addition, those among them who were in contact with follow-up services for other reasons also received oral and written information about the study. The study was conducted between March and June 2016. Youth contacts in the labor welfare system and community workers in the largest municipality assisted participants during the data collection period. A total of 105 respondents completed the survey and were included in the study.

#### The Young Data Study

In southern Norway, the Young Data study included adolescents that attended all junior high schools (13–16 years old) and first-year students in high school (16–17 years old). Data were collected from all 30 municipalities in this region (for more information on the Young Data study, see: https://www.ungdata.no/wp-content/uploads/2020/12/English-engelsk-Informasjonsskriv-VGS.pdf).

In addition, a strategic sample (based on geographic affiliation and study specialization) of 500 students that attended the second and third years of high school (17–19 years old) were recruited within the same geographical region. In total, 15,651 students were invited to participate during school h. The invitation was accepted by 11,042 (90%) of the junior high school students and 4,609 (80%) of the high school students. The present study only included the data reported by the high school students. Furthermore, the number of participants in the first year of high school was disproportionate to the numbers in the second and third years of high school; therefore, we performed individual matching to balance the sample, according to age.

To improve statistical power, it is recommended that the study should include controls and targeted participants at a ratio of approximately 4:1 (33). Thus, 96 respondents from the HELLAS study were matched, according to age and sex, with 384 respondents (controls) from the Young Data study (i.e., case: control, 1:4). Thus, the total sample size was 480 participants. The matching process was performed with the case-control matching procedure provided in SPSS software; the matching tolerance was set to zero.

### Measurement Instruments

Information about exposure to self-directed violence was retrieved by asking the following questions: “Have you ever tried to take your own life?” and “Have you ever tried to harm yourself?”. Response alternatives were “yes” and “no.”

Participants reported whether they had been exposed to violence from others by responding to the following three questions: “Have you ever been exposed to violent threats?”, “Have you ever been beaten without leaving visible marks?”, and “Have you ever been injured by an act of violence?”. The four response alternatives were: “never,” “once,” “two–five times,” and “six times or more.” Responses were dichotomized to “never” and “at least once.”

Information about the participant's sex was retrieved by asking participants in the Young Data study whether they were male or female. In the HELLAS study, an additional response alternative was included for respondents that defined themselves as something other than male or female. However, this latter option was not used in the present study, due to the low number of individuals in that group and the lack of that category in the Young Data study.

Parental educational levels were assessed by asking whether the participant's parent(s) had completed a university/college education.

In the HELLAS study, age was stratified in two-year age categories (16–17, 18–19, 20–21, and 22 years and older). Due to the low participation rate among the oldest age group, this group was merged with the 20–21-year age group. We implemented similar age categories (16–17 years, 18–19 years, and ≥20 years) in the Young Data study data to match participants according to age.

### Data Analysis

We performed *X*^2^-tests to evaluate differences in indicators of self-directed violence and violence from others between NEET/school groups and male/female groups (Table 1). Logistic regression analyses were stratified by sex and controlled for parental education. We examined potential associations between NEET status and exposure to self-directed violence and violence from others (Table 2). Results are reported as odds ratios (ORs) with 95% confidence intervals (CIs). The level of statistical significance was set to 5%. All analyses were conducted with IBM SPSS Statistics 25.0.

**Table 1:** Comparisons of violent experiences according to NEET status and sex.

**Variables**		**Females**			**Males**		
		**NEET (*n* = 43) *n* (%)**	**School students *(n* = 172) *n* (%)**	**p-value**	**NEET (*n* = 53) *n* (%)**	**School students (*n* = 212) *n* (%)**	**p-value**
Self-directed violence	Suicide attempt	19 (45.2)	32 (18.8)	<0.001	9 (17.6)	26 (13.1)	0.408
	Self-harm	33 (78.6)	57 (33.9)	<0.001	18 (34.6)	43 (21.8)	0.056
Violence from others	Experienced violent threats	13 (31)	13 (7.6)	<0.001	11 (21.6)	42 (20.7)	0.890
	Beaten without leaving visible marks	11 (26.2)	11 (6.5)	<0.001	7 (13.7)	21 (10.4)	0.498
	Injured by acts of violence	6 (14.3)	6 (3.6)	0.007	10 (20)	14 (6.9)	0.005

**Table 2:** Associations between NEET status and outcome variables, compared to high-school students (reference group), stratified by sex, and after adjusting for parental education.

		**Females**	**Males**
**Outcome variable**		**OR (CI 95%)**	**OR (CI 95%)**
**Suicide attempt**	**NEET^a^**	4.39 (1.96–9.85)^***^	0.80 (0.29–2.20)
	Low education, father^b^	1.61 (0.63–4.14)	0.71 (0.26–1.96)
	Low education, mother^b^	0.84 (0.33–2.16)	8.87 (2.29–34.31)^**^
**Self–harm**	**NEET^a^**	7.68 (3.20–18.41)^***^	1.51 (0.71–3.20)
	Low education, father^b^	0.74 (0.33–1.66)	1.60 (0.73–3.54)
	Low education, mother^b^	1.46 (0.65–3.30)	1.59 (0.76–3.48)
**Experienced violent threats**	**NEET^a^**	5.49 (2.06–14.66)^**^	0.61 (0.25–1.48)
	Low education, father^b^	1.48 (0.42–5.19)	1.20 (0.54–2.64)
	Low education, mother^b^	0.73 (0.20–2.57)	2.32 (1.04–5.19)^*^
**Beaten without visible marks**	**NEET^a^**	4.48 (1.58–12.72)^**^	1.46 (0.55–3.85)
	Low education, father^b^	1.31 (0.33–5.26)	1.19 (0.46–3.10)
	Low education, mother^b^	1.29 (0.31–5.27)	0.84 (0.33–2.17)
**Injured due to violence**	**NEET^a^**	6.77 (1.70–26.90)^**^	3.23 (1.20–8.62)^*^
	Low education, father ^b^	1.22 (0.19–7.73)	0.63 (0.21–1.88)
	Low education, mother ^b^	0.99 (0.15–6.76)	2.06 (0.65–6.58)

a *The NEET and control (school students) groups were matched for age*;

b *Compared to mothers/fathers with a higher education*.;

* *p < 0.05*

** *p < 0.01*

*** *p < 0.001*.

## Results

### Results From the Descriptive Analysis

Compared to males in upper-level secondary school, lower paternal education was more prevalent among NEET males (75.0 vs. 53.5 %, *p* < 0.007). Lower maternal education was more common among both NEET females compared to their female peers in school (73.8 vs. 51.9 %, *p* < 0.011) and among NEET males compared to their male peers in school (73.5 vs. 47.6 %, *p* < 0.001).

Further, descriptive statistics (Table 1) showed that, compared to females in upper-level secondary school, a higher number of females with NEET status reported suicide attempts (45.2 vs. 18.8%, *p* < 0.001) and self-harm (78.6 vs. 33.9%, *p* < 0.001). In contrast, indicators of self-directed violence were not significantly different between males with NEET status and males in upper-level secondary school.

Additionally, compared to females in upper-level secondary school, more females with NEET status had experienced violent threats (78.6 vs. 33.9%, *p* < 0.001), had been beaten without leaving visible marks (26.2 vs. 6.5%, *p* < 0.001), and had been injured due to violence (14.3 vs. 3.6%). Moreover, compared to males in upper-level secondary school, more males with NEET status had been injured due to violence (20 vs. 6.9%, *p* < 0.001), but these groups were not significantly different in other indicators of violence.

The correlation (Pearson's, two-tailed) between self-harm and suicide attempt was strong [*r*(456) = 0.59, *p* < 0.001]. The correlation was lower between self-ham and violent threats [*r*(450) = 0.25, *p* < 0.001], beaten without marks [*r*(448) = 0.23, *p* < 0.001], and injury due to violence [*r*(449) = 0.14, *p* < 0.002], and between suicide attempt and violent threats [*r*(452)=0.23, *p* < 0.001], beaten without marks [*r*(449) = 0.20, *p* < 0.001], and injury due to violence [*r*(450) = 0.22, *p* < 0.001].

### Results From Multivariable Logistic Regression

The multivariable logistic regression analysis, adjusted for parental education (Table 2), showed that females with NEET status had higher odds of self-directed violent behaviors, including suicide attempts (OR: 4.39; 95% CI: 1.96–9.85) and self-harm (7.68; 3.20–18.41), compared to females in upper-level secondary school. In contrast, these indicators of self-directed violence were not significantly different between males with NEET status compared to males in upper-level secondary school.

Furthermore, our results showed that females with NEET status had higher odds of experiencing violent behaviors from others, including violent threats (5.49; 2.06–14.66), beaten without leaving visible marks (4.48; 1.58–12.72), and injured due to violence (6.77; 1.70–26.90), compared to females in upper-level secondary school. Males with NEET status also had higher odds of being injured due to violence (3.23; 1.21–8.62) than males in upper-level secondary school, but these groups were not significantly different in other indicators of violence from others.

## Discussion

In this study, we described the prevalence of self-directed violence and violence from others among NEET young people, compared to peers that attended upper-level secondary school. Our results showed a prominent pattern among female participants. Overall, NEET females had much higher odds of reporting self-harm, suicide attempts, experience of violent threats, being beaten without visible marks, and being injured due to violence, compared to females that attended upper-level secondary school. These differences in self-directed violence and most types of violence from others were not evident among NEET males compared to males that attended upper-level secondary school. The exception was that NEET males had much higher odds of being injured due to violence, compared to their peers in school.

Our study indicated that the relationships between NEET status and the prevalence of self-directed violence and violence from others were strongly sex-dependent. This is in line with a pattern shown in other studies where the prevalence of self-harm is higher among females than males (15, 16) and with deliberate self-harm increasing over time more prominently among females than among males (19). Thus, Future studies are needed to identify potential causal effects between these factors and the mediating role of sex. One hypothesis that may explain a potential causal relationship between NEET status and self-harm is that NEET status constitutes a difficult life-situation that can induce mental health problems, which may lead to deliberate self-harm. Furthermore, consistent with results from the present study, we previously showed that the prevalence of mental health problems was higher among NEET females than among female peers in school. In contrast, the prevalence of mental health problems were similar among NEET males and their peers in upper-level secondary school (10). A potential explanation for the observed sex-related differences could be that girls internalize life difficulties to a greater degree than boys; thus, girls may be more likely to direct emotional pain toward themselves, and thus, self-harm may be a way of coping with emotional stress (18). However, over time, self-harm has slightly increased among boys (18); that finding suggested that factors other than sex may explain the increase in self-harm. One alternative explanation could be that the increase in self-harm is related to a rise in unemployment and marginalization from the job market. However, studies that investigated this hypothesis have reported inconsistent findings (23, 24).

Results from the present study also showed that NEET females, and to some extent, NEET males, had a higher risk of experiencing violence from others compared to their female and male counterparts in high school. This finding was consistent with findings from a registry-based study, which showed that victimization due to violence was more prevalent among unemployed female youths than among employed females (34). The WHO pointed out that, to meet the 2030 Sustainable Development Goals, violence in childhood should be recognized for its significant contribution to inequalities in education (4). Adverse experiences, such as violence during childhood, are socially patterned. Metzler et al. (35) found that participants that reported adverse experiences during childhood were more likely to report high-school non-completion and unemployment. Currie and Widom (36) conducted a prospective cohort study, where adults that experienced childhood abuse and/or neglect showed lower levels of education and employment. These results have also been confirmed by a registry-based study, which showed that individuals that experienced violence from others were excluded from the labor market more often than those that had not been exposed to violence from others (37).

Adverse childhood experiences, such as violence from others, may also incite negative emotions that impact self-harming behaviors, such as suicide attempts (4, 38). However, in the current study, the correlation between self-directed violence and violence from others was only low to moderate.

This study has provided valuable knowledge to extend the scarce previous research on the occurrence of self-directed violence and violence from others among NEET youths, compared to school-attending youths. Including NEET individuals in research has been difficult, because they comprise heterogenous group that is not gathered to a central organization, like school students; thus, they are less accessible for participating in studies. A strength of this study was the ability to target a previously registered vulnerable group of NEETs. However, the response rate limited a generalization of the results. All 695 eligible NEETs received written information about the study by mail, but only an unknown number were in touch with the advisory service to receive a face-to-face invitation. It is possible that the ones who came to advisory services and answered the questionnaire were the less vulnerable, and that the most marginalized youths stayed home. However, as the time of data collection in the present study coincided with application deadline for school attendance, the least vulnerable group of NEETs with intention of returning to school was most likely under-represented. The number of cases (NEETs) also limits the possibility to control for possible confounders such as mental health and substance use in risk of committing type II errors. Another limitation of the present study was the cross-sectional design, which restricted the possibility of analyzing causal inferences.

This study provided evidence indicating that young NEET girls were particularly vulnerable to self-directed violence and violence from others. Service providers within different sectors, such as the advisory service, school nurse, work related support services and pedagogic-psychological services, should be aware of the increased risk of violence and self-directed violence among NEETs compared to those in school. Thus, interventions should be developed to provide tailored support beyond pedagogic interventions or work-training, for example by including trauma-sensitive approaches to those who have been exposed to violence by others. This may imply cross-sectoral cooperation with mental health services.

## Data Availability Statement

The datasets presented in this article are not readily available because restrictions apply to the availability of these data. Data obtained from Ungdata are available at https://helsedata.no/en. Unfortunately, the participants recruited through the follow up services (NEET youths) have not consented to making their data available to a third party, and the researchers do not possess contact information for retrieving such consent. Requests to access the datasets should be directed to siri.h.haugland@uia.no or helsedata.no/en.

## Ethics Statement

The studies involving human participants were reviewed and approved by South-East Regional Committee for Medical and Health Research Ethics (REK case no. 2015/2431). Written informed consent from the participants' legal guardian/next of kin was not required to participate in this study in accordance with the national legislation and the institutional requirements.

## Author Contributions

Conceptualization and writing–original draft: SH and TS. Analysis: SH. Methodology and presentation of results: TS. All authors contributed to the article and approved the submitted version.

## Conflict of Interest

The authors declare that the research was conducted in the absence of any commercial or financial relationships that could be construed as a potential conflict of interest.

## Publisher's Note

All claims expressed in this article are solely those of the authors and do not necessarily represent those of their affiliated organizations, or those of the publisher, the editors and the reviewers. Any product that may be evaluated in this article, or claim that may be made by its manufacturer, is not guaranteed or endorsed by the publisher.
